# A. V. M. A.

**Published:** 1902-04

**Authors:** 


Secretary.

---

### A. V. M. A.

### MINNEAPOLIS, MINN., SEPTEMBER, 1902.

Drs. Lyford and Brimhall, of Minneapolis, were in attendance at the winter meeting of the Iowa State Veterinary Association in the interest of the coming meeting of the A. V. M. A. in Minneapolis. They give the Iowa people credit for having an effective, hard-working association, and report a good time for themselves.

A committee was appointed by the Iowa Association for the purpose of getting Iowa veterinarians to attend the meeting of the International Association in the largest possible numbers. They will co-operate with the resident State secretary in this good work of insuring a record-breaking attendance from their State.

Every sign points toward a meeting of the A. V. M. A. in Minneapolis this fall that will be historical.

Drs. Reynolds and Brimhall attended the annual meeting of the Wisconsin State Association in the interests of the big meeting of the A. V. M. A. in Minneapolis this fall. The Wisconsin men have already taken steps to insure a good attendance from their State. A committee was appointed to assist the resident State secretary in this good work of keeping the meeting before the attention of the profession in that State.

